# A New Approach Using Autologous Platelet-Rich Plasma to Treat Infertility and To Improve Population Replacement Rate 

**Published:** 2016-09-20

**Authors:** Marzie Farimani, Maryam Bahmanzadeh, Jalal Poorolaja

**Affiliations:** ^a^ Endometrium and Endometriosis Research Center, Hamadan University of Medical Sciences, Hamadan, Iran; ^b^ Department of Epidemiology, School of Public Health, Hamadan University of Medical Sciences, Hamadan, Iran; ^c^ Research Center for Health Sciences, Hamadan University of Medical Sciences, Hamadan, Iran

## Dear Editor-in-Chief

The total fertility rate (TFR) or the number births over a woman's lifetime, has reached 1.8 in 2012 which is the lowest among Islamic countries, and even below the world average of 2.1 births per woman^1-2^. This is less than population replacement rate. Furthermore, increasing age of marriage and change in lifestyle such as exposure to environment toxins increased the incidence rate of infertility^3^. More than three million people and nearly 15% of couples suffer from infertility^4^. This dilemma results in adverse economic consequences and physical, mental, and emotional problems to families. Despite progress made in the field of assisted reproductive technology (ART), multiple embryo failed to implant. A significant percentage of In Vitro Fertilization (IVF) failure is due to the endometrial receptivity^5^. Implantation requires to good quality embryo to provide a good coordination between mother and fetus. Human endometrium undergoes significant changes during implantation and immune cells and their secreted substances such as granulocyte colony-stimulating factor (G-CSF) in the luteal phase play an important role^6^.

In an effort to increase the thickness of endometrium, several approaches have been made, but the results are still questionable. Platelets contain a significant amount of growth factors^7^ that have positive effects on local tissue repair ^8^. Larson et al., successfully developed bovine embryos during the fourth cell cycleusing platelet-derived growth factor (PDGF)^9^. This was the beginning of an evolution in reducing abortion rate by increasing endometrial thickness. Thereafter, further animal and human studies were conducted to demonstrate the efficacy of PDGF administration for infertility.

Chang et al. administered intrauterine infusion of platelet rich plasma (PRP) in infertile women with thin endometrium and reported good results 4 pregnancy from five patients with thin endometrium and poor response to conventional therapy during freeze embryo transfer^10^. PRP is quite a new treatment used for the improvement the endometrial thickness in women with thin endometrium. The use of PRP is considered safe because of autologous nature derived from patient’s own blood^11^.

In 2016, for the first time, we performed a single-blind pilot study in Hamadan, western Iran to explore the hypothesis that intrauterine administration of PRP could improve pregnancy outcome of frozen–thawed embryo transfer in nine infertile women with a history of recurrent implantation failure (RIF) who had failed to achieve a clinical pregnancy which at least three or more good quality embryos transfers (Table 1). Hormone replacement therapy was carried out and then 0.5-1 mL PRP was introduced to the uterine cavity under ultrasound guidance using Wallace catheter about 36 h before undergoing frozen embryo transfer. We selected the embryos for transfer at morula stage. A clinical pregnancy was confirmed by blood βHCG, 14 d after embryo transfer. The mean number of embryo transfer cycle and ART cycle was 3.3 and 2, respectively. The mean number of embryo transfer in current cycle was 2.5 embryos. Six women achieved clinical pregnancy and the mean pregnancy rate was 66.6%. All pregnancies are in progress normally. The gestational age of first ongoing pregnancy was 26 weeks.

**Table 1:** Characteristics of the study population

**Women**	**Age** **(yr)**	**Infertility** **status**	**Infertility** **cause**	**Previous** **pregnancy**	**Duration of** **infertility (yr)**	**No. of embryo transfer**		**Clinical** **Pregnancy**	**Gestational** **Age (week + day)**
						**Before**	**Current**		
1	35	Primary	Male	Negative	11.0	3	3	Negative	-
2	36	Secondary	Male & Female	Positive	4.5	3	1	Positive	8 + 0
3	33	Primary	Male & Female	Negative	12.0	5	2	Positive	5 + 1
4	35	Primary	Male & Female	Negative	1.5	8	3	Positive	14 + 0
5	45	Primary	Female	Negative	5.0	12	3	Positive	28 + 4
6	40	Secondary	Female	Positive	6.5	8	2	Positive	7 + 4
7	36	Primary	Female	Negative	1.0	4	2	Negative	-
8	30	Primary	Male	Negative	3.0	10	3	Negative	-
9	39	Secondary	Male	Positive	2.0	3	3	Positive	8 + 0

Despite limitations typical to a pilot study, including a small sample size and lack of a control group, this pilot study suggests that PRP administration before embryo transfer may play a vital role in successful implantation. This study is in progress and we will report the final results subsequently.

This study was conducted for the first time at the Endometrium and Endometriosis Research Center, Hamadan University of Medical Sciences, Hamadan, the west of Iran, from April to July 2016. Written informed consent was received from all parents. The Ethics Committee of the university approved the study (IR.UMSHA.REC.1395.272). The protocol was registered with the Iranian Registry of Clinical Trials (IRCTIRCT201608319014N113).

## Acknowledgement

The authors thank the staff of the Hamadan Blood Transfusion Organization and Endometrium and Endometriosis Research Center for their valuable collaboration with this work. The authors declare that there is no conflict of interest.

Marzie Farimani (MD)^a^, Maryam Bahmanzadeh (PhD)^a^ , Jalal Poorolajal (MD, PhD)^bc^*

^a^ *Endometrium and Endometriosis Research Center, Hamadan University of Medical Sciences, Hamadan, Iran*

^b^ *Department of Epidemiology, School of Public Health, Hamadan University of Medical Sciences, Hamadan, Iran*

^c^ *Research Center for Health Sciences, Hamadan University of Medical Sciences, Hamadan, Iran*

*****Correspondence to:***** *Jalal Poorolajal (MD, PhD)*

**E-mail:** poorolajal@umsha.ac.ir

## References

[1] Mihigo R, OMalley H, Chaudhri I (2015). World fertility patterns 2015. Stud Fam Plann.

[2] Kazemijaliseh H, Behboudi-Gandevani S, Hosseinpanah F, Khalili D, Azizi F (2015). The prevalence and causes of primary infertility in Iran: a population-based study. Glob J Health Sci.

[3] Marshburn PB (2015). Counseling and diagnostic evaluation for the infertile couple. Obstet Gynecol Clin North Am.

[4] Sanchez E, Giviziez CR, Sanchez HM, Agostinho P, Barros PS, Approbato MS (2015). Low progesterone levels and ovulation by ultrasound assessment in infertile patients. J Bras Reprod Assist.

[5] Zeyneloglu HB, Onalan G (2014). Remedies for recurrent implantation failure. Semin Reprod Med.

[6] Toth B, Würfel W, Germeyer A, Hirv K, Makrigiannakis A, Strowitzki T (2011). Disorders of implantation–are there diagnostic and therapeutic options?. J Reprod Immunol.

[7] Macaulay IC, Carr P, Gusnanto A, Ouwehand WH, Fitzgerald D, Watkins NA (2005). Platelet genomics and proteomics in human health and disease. J Clin Invest.

[8] Rudkin GH, Miller TA (1996). Growth factors in surgery. Plast Reconstr Surg.

[9] Larson R, Ignotz G, Currie W (1992). Platelet derived growth factor (PDGF) stimulates development of bovine embryos during the fourth cell cycle. Development.

[10] Chang Y, Li J, Chen Y, Wei L, Yang X, Shi Y, Liang X (2015). Autologous platelet-rich plasma promotes endometrial growth and improves pregnancy outcome during in vitro fertilization. Int J Clin Exp Med.

[11] Zwiep T, Humphrey R, Fortin D, Inculet RI, Malthaner RA (2016). Autologous platelet rich plasma and concentrated platelet poor plasma are safe in patients requiring lobectomies but do not reduce the duration of air leak: a randomized controlled trial. Ann SurgInt.

